# Effect of xanthan gum-based food thickeners on the dissolution profile of fluoroquinolones oral formulations

**DOI:** 10.1186/s40780-020-00181-9

**Published:** 2020-11-30

**Authors:** Nobuyuki Takahashi, Yoshiaki Fujita, Nanako Takahashi, Akihiro Nakamura, Tsutomu Harada

**Affiliations:** 1 Division of Pharmaceutics, Department of Pharmacology, Toxicology and Therapeutics, School of Pharmacy, Showa University, 1-5-8, Hatanodai, Shinagawa-ku, Tokyo, 142-8555 Japan; 2 Department of Hospital Pharmaceutics, School of Pharmacy, Showa University, Tokyo, Japan

**Keywords:** Fluoroquinolone, Xanthan gum, Film coating, Ciprofloxacin, Food thickener, Dissolution test, Dysphagia

## Abstract

**Background:**

Xanthan gum-based food thickeners (XG-FTs) are often ingested by patients with dysphagia to prevent aspiration during drug treatment. Reportedly, XG-FTs affect tablet disintegration, drug dissolution rates, and reduce the efficacy of postprandial antihyperglycemic agents. The absorption rate and quantity of fluoroquinolone antimicrobial agents correlate with drug efficacy, raising concern about the impact of XG-FTs. Previously, we reported that film-coated tablets were less susceptible to the effects of XG-FT than conventional and orally disintegrating tablets. Here, we compare the effect of XG-FTs on dissolution profiles of three oral fluoroquinolone-based film-coated tablets by evaluating the dissolution of crushed products, fine granules, and film-coated fine granules.

**Methods:**

We examined formulations of tosufloxacin tosylate monohydrate (TFLX), levofloxacin hemihydrate (LVFX), and ciprofloxacin hydrochloride hydrate (CPFX). The formulations were immersed in 20 mL of 1.5% (w/v) XG-FT aqueous solution for 2.5 min followed by a dissolution test using the paddle method according to the Japanese Pharmacopoeia (dissolution test solution pH 1.2; volume 900 mL; temperature 37 ± 0.5 °C). The dissolution profile was evaluated according to the dissolution quantity indicated in product specifications and guidelines for bioequivalence testing of generic drugs. The 15-min mean dissolution rate was determined for a formulation immersed in 1.5% (w/v) XG-FT aqueous solution and compared with that for a non-immersed formulation (control). Fluoroquinolone film-coated tablets were mixed with starch-based FTs, guar gum-based FTs, or XG-FTs to observe their appearances.

**Results:**

The dissolution profile of LVFX film-coated tablets was not affected by XG-FTs, but the dissolution of TFLX and CPFX was delayed. For crushed film-coated tablets, the 15-min mean dissolution rate was significantly delayed for all three fluoroquinolones when compared with that of uncrushed products. The dissolution profile of TFLX film-coated fine granules was unchanged by XG-FTs. CPFX film-coated tablets and crushed products produced a gel-like precipitate when mixed with XG-FTs and failed to meet product-dissolution specifications. A gel-like precipitate was also observed with guar gum-based FTs.

**Conclusion:**

The effect of XG-FTs on the dissolution profile of film-coated fluoroquinolone formulations varied depending on the formulation. The CPFX formulation formed a gel-like precipitate when immersed in XG-FTs resulting in a significantly delayed dissolution.

## Background

Food thickeners (FTs) are often used by patients with dysphagia to prevent aspiration and are classified into three types (starch, guar gum, and xanthan gum) based on the polysaccharide type. The third-generation xanthan gum-based FTs (XG-FTs) are most frequently used as they readily dissolve without impairing the product taste. FTs are mainly used to control the viscosity of beverages and the cohesiveness of boluses to prevent aspiration. They are often utilized in elderly individuals owing to the high prevalence of dysphagia. Based on the increased mortality from suffocation and aspiration pneumonia, the use of FTs is important in elderly persons to prevent aspiration. In Japan, the use of XG-FTs has been reported in 34 of 36 nursing-care facilities surveyed, indicating that XG-FTs may be used together with the administration of pharmaceutical products [1]. The use of FTs for delivering medications has been indicated for managing oral drug administration in patients with dysphagia [2].

It has been reported that XG-FTs affect tablet disintegration and dissolution, delaying the release of drugs such as mitiglinide calcium hydrate, donepezil hydrochloride, and levofloxacin hydrate [3, 4]. XG-FTs delay the dissolution of effervescent acetaminophen tablets by more than 3 h [5]. The hypoglycemic effect of conventional mitiglinide calcium hydrate and orally disintegrating voglibose tablets is reportedly inhibited by XG-FTs [6, 7].

Oral formulations are classified by the biopharmaceutics classification system (BCS) according to the solubility and membrane permeability of the active pharmaceutical ingredient. BCS is one criterion for extrapolating in vitro data to assess bioequivalence [8]. For example, fluoroquinolone antibacterial agents demonstrate high membrane permeability [9]. The absorption rate of a drug with a high membrane permeability is greatly affected by the drug dissolution rate. The antimicrobial activity of fluoroquinolones correlates with the maximum serum concentration and the area under the curve of the drug concentration-time profile [10]. A delay in the dissolution of fluoroquinolones leads to a decrease in the released-drug quantity and rate of absorption and might reduce drug efficacy. Fluoroquinolones have broad antibacterial activity, excellent tissue penetration, and are clinically important antibacterial agents. Our laboratory previously reported that the disintegration and dissolution of film-coated tablets were less affected by XG-FTs than those of orally disintegrating tablets [3]. It appeared that the combined use of XG-FTs and oral agents was least likely to be affected by prolonged disintegration time and delayed dissolution when a film coating was applied. In the present study, we investigated the effect of XG-FTs on the dissolution profiles of levofloxacin hemihydrate (LVFX), tosufloxacin tosylate monohydrate (TFLX) tablets, and ciprofloxacin hydrochloride hydrate (CPFX). LVFX is the most frequently used fluoroquinolones in Japan [11]. TFLX is used at a wide range of ages as pediatric fine granules are approved in Japan. CPFX is highly effective against *Pseudomonas aeruginosa* among the fluoroquinolones [12].For all three fluoroquinolone tablets, hypromellose was used as the film-coating agent. Crushed film-coated tablets were used for comparison to mimic the damage to the film coating. We also investigated the effects of XG-FTs in the absence or presence of a coating on fine granules.

## Material and methods

We investigated TFLX, LVFX, and CPFX film-coated tablets. Hypromellose was used as the film-coating agent. The crushed product was prepared by wrapping tablets in medicinal wrapping paper and crushing using a pestle. The change in weight before and after crushing was within 1%. For TFLX, film-coated fine granules and uncoated fine granules were also evaluated. The components present in each investigated fluoroquinolone-based formulation are listed in Table 1.

**Table 1:** Excipients in fluoroquinolone-based formulations

Dosage form	TFLX			LVFX	CPFX
	Tablets	Uncoated fine granules	Film-coated fine granules	Tablets	Tablets
Film coating agent	Hypromellose	None	Polyvinyl alcohol/acrylic acid/methyl methacrylate copolymer	Hypromellose	Hypromellose
Other	L-aspartic acid, crystalline cellulose, corn starch, hydrous silicon dioxide, hydroxypropylcellulose, magnesium stearate, polyoxyethylene (105) polyoxypropylene (5) glycol, talc, titanium oxide, carnauba wax	Sucrose, aspartame, hydroxypropylcellulose, hydrous silicon dioxide, iron sesquioxide, fragrance	Refined sucrose, L-aspartic acid, aspartame, iron sesquioxide, hydrous silicon dioxide, fragrance	Crystalline cellulose, carboxymethyl cellulose, hydroxypropyl cellulose, sodium stearyl fumarate, titanium oxide, talc, macrogol 6000, yellow ferric oxide, iron sesquioxide, carnauba wax	Crystalline cellulose, corn starch, crospovidone, light anhydrous silicic acid, magnesium stearate, macrogol 4000, titanium oxide

### Preparation of samples

Tsururinko Quickly^Ⓡ^ (Clinico, Tokyo, Japan) was dissolved in purified water and allowed to stand at room temperature for 30 min to obtain a 1.5% (w/v) solution of XG-FTs. Tuning-fork viscometer SV-10A (A&D Company, Tokyo, Japan.) was one-point calibrated using the viscometer-calibration standard solution JS50 (Nippon Grease Co., Kanagawa, Japan.) showing its viscosity after 3 min at a constant temperature of 20 ± 0.5 °C to be in the range of 50–150 mPa·s. The mean viscosity of the 1.5% (w/v) XG-FT was 104.2 ± 17.4 mPa·s (*N* = 3). This viscosity corresponded to the category of mildly thick viscous products according to the classification for swallowing-adjusted foods by the Japanese Society of Dysphagia Rehabilitation 2013 [13].

The Japanese Pharmacopoeia (JP) first fluid (10 mL) or 1.5% (w/v) XG-FT was placed in a polystyrene cup (90 mL volume). A fluoroquinolone formulation or crushed film-coated tablet was added to the cup together with an additional 10 mL of the corresponding fluid. Samples of fine granules or crushed film-coated tablets were prepared using the above liquids and mixed 10 times with a teaspoon. The preparation of each sample took approximately 2.5 min. Each sample (20 mL; total of six samples) was added to the dissolution-tester vessel, and the volume was adjusted to 900 mL. Formulations not immersed in 1.5% (w/v) XG-FT were used as controls.

### Dissolution test

The paddle method (50 rpm, JP first fluid pH 1.2, 900 mL, 37 ± 0.5 °C) was employed according to the JP guidance using the dissolution tester NTR-6400A (Toyama Sangyo Co., Osaka, Japan). Aliquots were sampled at 5, 15, 30, 60, and 90 min using the autosampler SAS-6000 (Toyama Sangyo Co. Osaka, Japan). Briefly, a 20-mL sample was collected by microfiltration through filter F-72 (Toyama Sangyo Co. Osaka, Japan); 10 mL volume was discarded. JP first fluid (20 mL) was added to the dissolution vessel. Product specifications were based on the Japanese Pharmaceutical Quality Information Collection. The desired dissolution rate was defined as 80% or more at 90 min for LVFX tablet, 65% or more at 90 min for TFLX, and 85% or more at 15 min for CPFX. Dissolution profiles were evaluated against controls according to the bioequivalence guidelines for fluoroquinolone-based formulations immersed in 1.5% (w/v) XG-FT [14]. When the average dissolution profiles of the control reached 85% within 15 min, the average dissolution within 15 min of LVFX crushed film-coated tablets, TFLX fine granules, CPFX film-coated tablets, and crushed film-coated tablets were considered similar if the dissolution was ±15% of that of the control (D15min). Fluoroquinolones that demonstrated an average dissolution rate similar to that of controls passed the test. Samples that did not meet the dissolution condition of at least 85% during 15–30 min were considered similar to controls using the f2 function. For TFLX film-coated and crushed film-coated tablets, the average dissolution rates were assessed at 5 and 30 min, respectively, with the average dissolution rates of the standard preparation being approximately 40 and 85%, respectively. For LVFX film-coated tablets, the average dissolution rate was determined at 15 and 30 min. The results showed the value of the f2 function to be ≥42.

### Absorbance measurement

Using the spectral scanning multimode reader VarioSkan Flash (Thermo Fisher Scientific Inc. Massachusetts, USA.), TFLX presented peak at the absorption wavelength of 346 nm, LVFX at 289 nm, and CPFX at 316 nm. TFLX with 95% chemical purity (FUJIFILM Wako Pure Chemical Corporation, Osaka, Japan.), LVFX with 98% chemical purity (FUJIFILM Wako Pure Chemical Corporation, Osaka, Japan.), and CPFX hydrochloride monohydrate with 98% chemical purity (FUJIFILM Wako Pure Chemical Corporation, Osaka, Japan.) were used to plot absorbance versus concentration calibration curve used to determine measurement linearity. The measured concentration range corresponded to the dissolution rate of 25–100% for each drug. All calibration curves were analyzed by regression analysis as R2 ≤ 0.97.

### Appearance observation after mixing film-coated fluoroquinolone tablets with FTs

To observe the appearances, a 1.5% (w/v) solution of XG-FT was prepared and mixed with film-coated fluoroquinolones tablets or crushed film-coated fluoroquinolones tablets in the same manner as samples for dissolution testing. Additionally, 1.5% (w/v) aqueous solutions of the starch-based FT, Tromelin^Ⓡ^ granules (NUTRI Co. Mie, Japan.), or guar gum-based FT, Toromi up ace^Ⓡ^ (Nisshin OilliO Co., Tokyo, Japan), were prepared and mixed with crushed film-coated fluoroquinolone tablets. The crushed film-coated tablets were mixed well with FTs and observed for their appearances.

### Statistical analysis

Based on bioequivalence guidelines, D15min values were subjected to statistical analysis to determine the dissolution rate that could be compared at the earliest time. The dissolution rate is expressed as the mean dissolution rate ± standard deviation. Mean dissolution rates were compared using the unpaired t-test or Welch test after evaluating the variance by Levene’s test. Data were analyzed using SPSS Statistics ver. 25 (IBM Corp., Armonk, NY, USA). The significance level was set at *p* < 0.05.

## Results

### Effect of XG-FTs on the dissolution of film-coated fluoroquinolone formulations

**Fig. 1:**
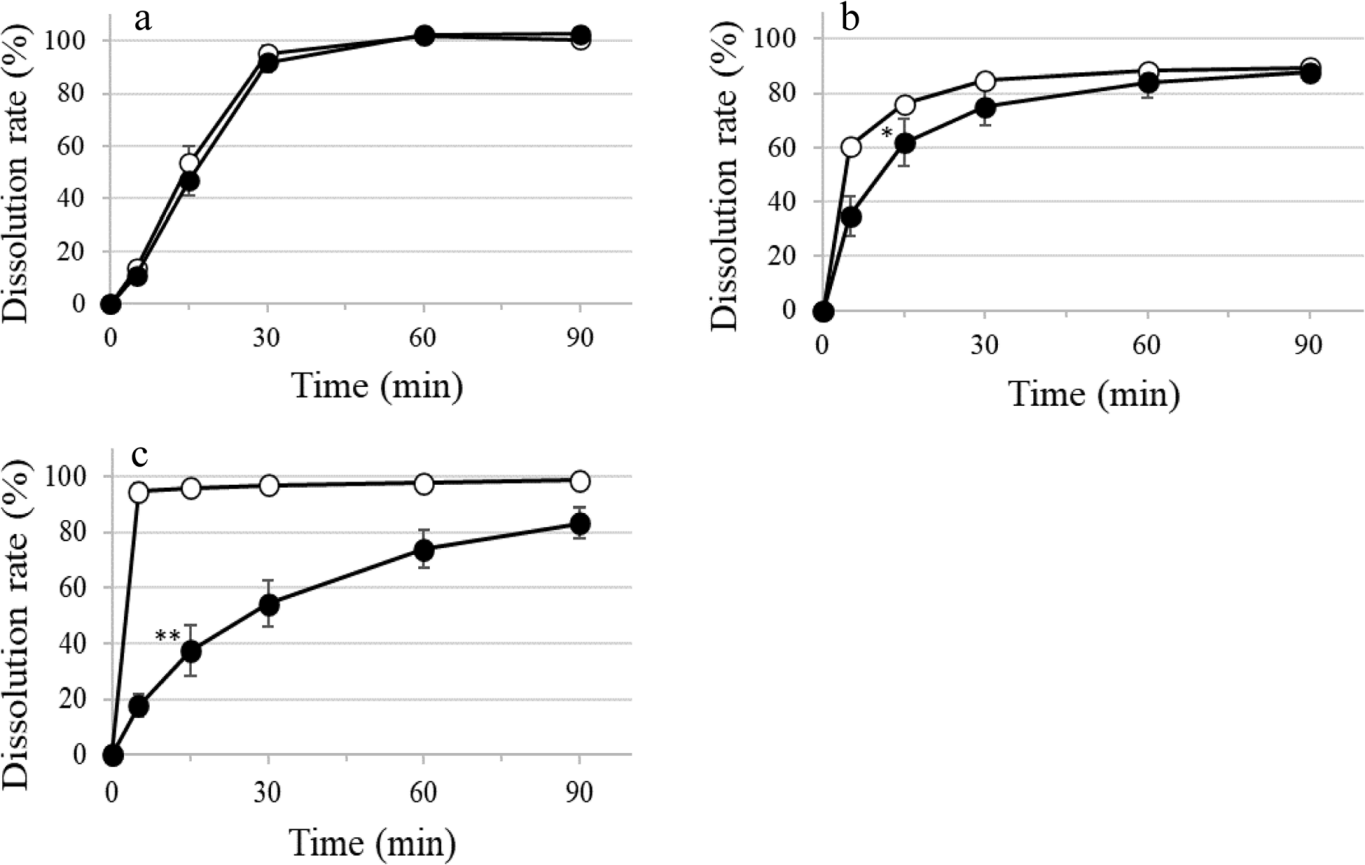
Effect of 1.5% (w/v) XG-FTs on the dissolution of fluoroquinolone film-coated tablets. ○ Control; ● immersed in 1.5% (w/v) XG-FTs; **a** LVFX film-coated tablets, **b** TFLX film-coated tablets, **c** CPFX film-coated tablets. Paddle method (solution pH 1.2; volume 900 mL; temperature 37 ± 0.5 °C, 50 rpm). TFLX film-coated tablets D15min; Welch test. LVFX or CPFX film-coated tablets D15min; unpaired *t*-test: *P < 0.05, **P < 0.01 vs. Control, *n* = 6, mean ± SD

Film-coated LVFX tablets immersed in 1.5% (w/v) XG-FTs conformed with product specifications as the mean dissolution rate exceeded 80% at 90 min. The mean dissolution rate at 15 min and 30 min presented an f2 value of 64 when compared with the control. Because the f2 value was above 42 (Fig. 1a), the similarity was confirmed. Delayed dissolution was observed with TFLX and CPFX film-coated tablets due to the effect of XG-FTs. It took 60 min for the dissolution of TFLX film-coated tablets to be within 5% difference from the control values, but the mean dissolution rate at 90 min was 65% or higher, thus meeting product specifications (Fig. 1b). Because the f2 value of the dissolution profile compared with the control was 35, the TFLX tablets were not considered similar; D15min was significantly decreased. CPFX film-coated tablets showed 98.7% dissolution within 5 min of the control, but D15min was 49.2% owing to the influence of XG-FTs, thus failing to conform with product specifications (Fig. 1c). Because the D15min value was not within 15% of the control, a significant decrease was observed that did not satisfy the similarity criterion. A large difference was observed in the dissolution profile of film-coated fluoroquinolone tablets although the same coating agent was used.

**Fig. 2:**
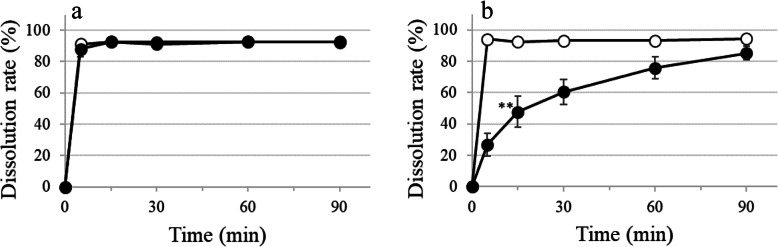
Effect of 1.5% (w/v) XG-FTs and film coating on the dissolution of fluoroquinolone fine granules. ○ Control; ● fine granules immersed in 1.5% (w/v) XG-FTs. **a** TFLX film-coated fine granules, **b** TFLX-uncoated fine granules. Paddle method (solution pH 1.2; volume 900 mL; temperature 37 ± 0.5 °C; 50 rpm). TFLX film-coated fine granules, D15min; unpaired *t*-test. TFLX film-uncoated fine granules, D15min; Welch test: *P < 0.05, **P < 0.01 vs. Control, *n* = 6, mean ± SD

Fine granules of film-coated TFLX immersed in 1.5% (w/v) XG-FTs satisfied product specifications, exhibiting a D15min value that did not significantly differ from the control (Fig. 2a). The dissolution profile of uncoated TFLX fine granules demonstrated a mean dissolution of 92.4% at 15 min. However, D15min was 47.7% due to the influence of XG-FTs. D15min was significantly delayed when compared with the control; the dissolution profiles lacked similarity. But the TFLX product specifications were satisfied.

### Effect of XG-FTs on the dissolution of crushed film-coated fluoroquinolone formulations and appearances of the dissolution test sample

**Fig. 3:**
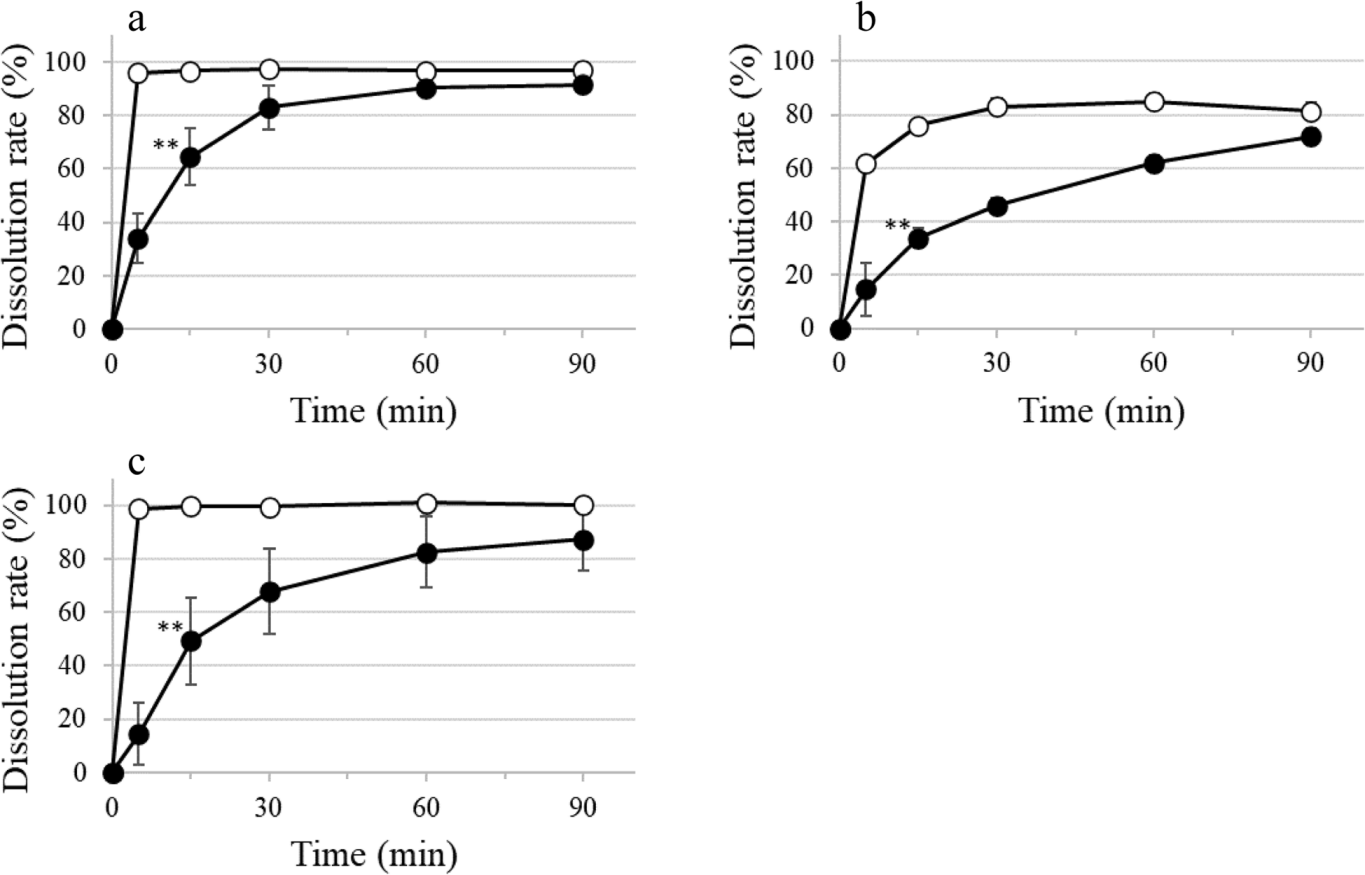
Effect of 1.5% (w/v) XG-FTs on the dissolution of fluoroquinolone crushed film-coated tablets. ○ Control; ● immersed in 1.5% (w/v) XG-FTs. **a** LVFX crushed film-coated tablets, **b** TFLX crushed film-coated tablets, **c** CPFX crushed film-coated tablets. Paddle method (solution pH 1.2; volume 900 mL; temperature 37 ± 0.5 °C; 50 rpm). LVFX or CPFX crushed film-coated tablets, D15min; Welch test. TFLX crushed film-coated tablets, D15min; unpaired *t*-test: *P < 0.05, **P < 0.01 vs. Control, *n* = 6, mean ± SD

For crushed film-coated LVFX tablets immersed in 1.5% (w/v) XG-FTs, the D15min was significantly lower than that of the control, and the dissolution profile lacked similarity (Fig. 3a). For crushed film-coated TFLX tablets immersed in 1.5% (w/v) XG-FTs, the dissolution profile showed no similarity as the f2 value was 19 when compared with the control, and D15min was significantly decreased (Fig. 3b). For the D15min value of crushed film-coated CPFX tablets immersed in 1.5% (w/v) XG-FTs, variation in the extent of dissolution was increased by crushing while dissolution was significantly decreased when compared with the control (Fig. 3c).

**Fig. 4:**
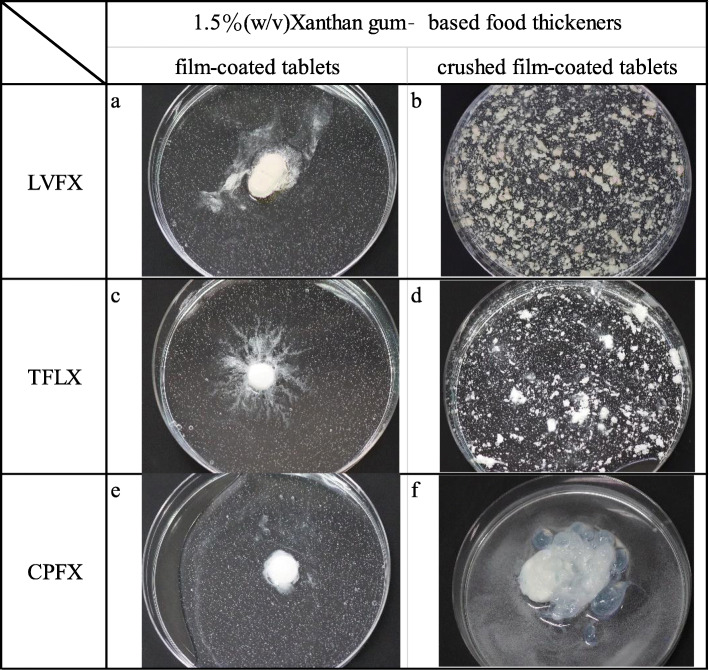
Appearances of crushed fluoroquinolone film-coated tablets mixed with 1.5% (w/v) XG-FTs. **a** LVFX film-coated tablets immersed in 1.5% (w/v) XG-FTs, **b** LVFX crushed film-coated tablets immersed in 1.5% (w/v) XG-FTs, **c** TFLX film-coated tablets immersed in 1.5% (w/v) XG-FTs, **d** TFLX crushed film-coated tablets immersed in 1.5% (w/v) XG-FTs, **e** CPFX film-coated tablets immersed in 1.5% (w/v) XG-FTs, **f** CPFX crushed film-coated tablets immersed in 1.5% (w/v) XG-FTs. CPFX film-coated tablets did not disintegrate owing to the gel layer formed on the surface (**e**). However, crushed CPFX film-coated tablets separated into a liquid and a translucent gel (**f**). Crushed LVFX and TFLX film-coated tablets were non-uniformly dispersed following XG-FT addition (**a**, **b**, **c**, **d**)

Film-coated LVFX and TFLX tablets immersed in 1.5% (w/v) XG-FTs presented a disintegrated surface (Fig. 4a, c). However, for CPFX immersed in 1.5% (w/v) XG-FTs, a semi-transparent gel layer was formed on the surface of film-coated tablets (Fig. 4e). For crushed products mixed with 1.5% (w/v) XG-FTs, LVFX and TFLX were non-uniformly dispersed (Fig. 4b, d), whereas CPFX formed a gel-like precipitate of decreased liquid viscosity (Fig. 4f).

### Appearances of starch or guar gum-based FTs mixed with crushed film-coated fluoroquinolones tablets

**Fig. 5:**
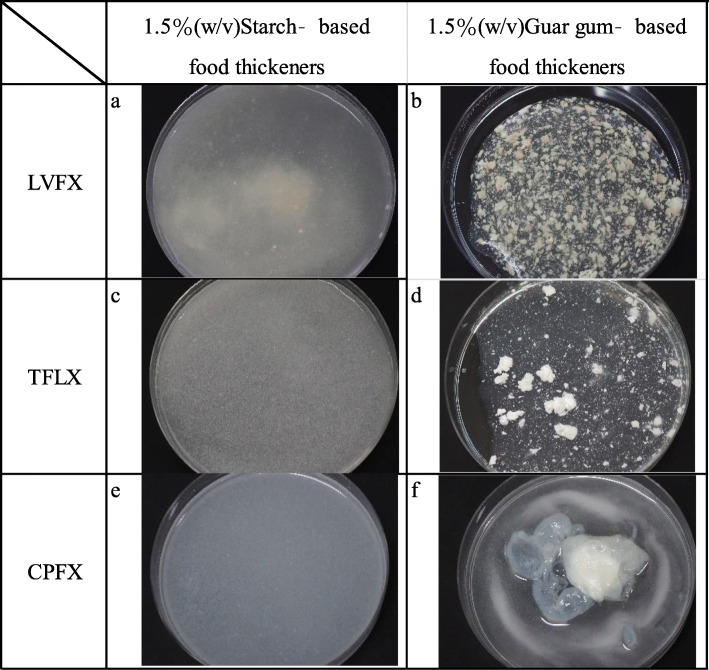
Appearances of crushed fluoroquinolone film-coated tablets mixed with 1.5% (w/v) starch- or guar-gum-based FTs. **a** LVFX crushed film-coated tablets immersed in 1.5% (w/v) starch-based FTs, **b** LVFX crushed film-coated tablets immersed in 1.5% (w/v) guar-gum-based FTs, **c** TFLX crushed film-coated tablets immersed in 1.5% (w/v) starch-based FTs, **d** TFLX crushed film-coated tablets immersed in 1.5% (w/v) guar-gum-based FTs, **e** CPFX crushed film-coated tablets immersed in 1.5% (w/v) starch-based FTs, **f** CPFX crushed film-coated tablets immersed in 1.5% (w/v) guar-gum-based FTs. Crushed fluoroquinolone film-coated tablets mixed with starch-based FTs showed uniform dispersion (**a**, **c**, **e**). Fluoroquinolone film-coated tablets mixed with guar gum-based FTs showed appearances similar to those mixed with XG-FTs (**b**, **d**, **f**)

Crushed film-coated fluoroquinolone tablets mixed with 1.5% (w/v) starch-based FTs were more evenly dispersed than when mixed with 1.5% (w/v) XG-FTs (Fig. 5a, c, e). LVFX and TFLX crushed film-coated tablets mixed with 1.5% (w/v) guar gum-based FTs were more unevenly dispersed than when mixed with 1.5% (w/v) starch-based FTs (Fig. 5b, d, f). CPFX crushed film-coated tablets immersed in 1.5% (w/v) guar gum-based FTs formed a gel-like precipitate similar to that observed when mixed with 1.5% (w/v) XG-FTs.

## Discussion

This study has shown the effect of film coating and fluoroquinolones (drug) on dissolution delay induced by XG-FTs. Since film-coated CPFX tablets interacted with XG-FTs resulting in a significant decrease in the mean dissolution rate, we highlighted factors that should be considered when using XG-FTs for the delivery of medications.

The effect of XG-FTs on the dissolution of hypromellose film-coated fluoroquinolone formulations varied depending on the formulation. For film-coated LVFX tablets, the dissolution remained unaffected by XG-FTs (Fig. 1a) but was delayed when the film coating was broken by crushing (Fig. 3a). With film-coated TFLX tablets, delayed dissolution was observed owing to the influence of XG-FTs (Table 2). The D15min of film-coated TFLX tablets immersed in 1.5% (w/v) XG-FTs was significantly reduced and the dissolution profiles lacked similarity; however, product specifications were satisfied (Fig. 1b). Breaking the surface of the film-coated TFLX tablets by crushing increased the dissolution delay induced by XG-FTs (Fig. 3b). Conversely, film-coated TFLX fine granules were not affected by XG-FTs (Fig. 2). In a previous study, we reported that the effect of XG-FTs on the dissolution delay was smaller in film-coated tablets than in conventional tablets [3]. We showed that the film coating of formulations reduced dissolution delays attributed to XG-FTs but the effect varied depending on the formulation. XG-FTs comes first into contact with the coating film, and contact with the dosage-formulation core is less likely to occur; hence, the effect on dissolution delay is reduced.

**Table 2:** Dissolution-rate conformance and similarity of fluoroquinolone-based formulations containing 1.5% (w/v) XG-FT with the reference

Dosage form	TFLX				LVFX		CPFX	
	Tablets	Crushed tablets	Uncoated fine granules	Film-coated fine granules	Tablets	Crushed tablets	Tablets	Crushed tablets
Conformance to specification	Yes	Yes	Yes	Yes	Yes	Yes	No	No
Judgement of similarity	No	No	No	Yes	Yes	No	No	No

In contrast, CPFX formulations were substantially affected by dissolution delays owing to XG-FTs regardless of the presence or absence of a film coating. Film-coated CPFX tablets immersed in 1.5% (w/v) XG-FTs failed to satisfy product specifications and similarity of dissolution profiles (Table 2). This may be attributed to the formation of a gel-like precipitate following the interaction between XG-FTs and CPFX film-coated tablets (Fig. 4e, f).In this study, we clarified that mixing CPFX formulations with an aqueous solution of XG-FTs induces a clinically relevant dissolution delay. The mixing of CPFX formulations with FTs should be undertaken cautiously since the CPFX formulation produced a similar gel-like precipitate when mixed with an aqueous solution of a guar gum-based FT (Fig. 4f). Excipients contained only in CPFX film coated tablets are light anhydrous silicic acid and crospovidone. However, since both are insoluble, interaction with xanthan gum is unlikely. It is necessary further to investigate interactions between drug substance and polysaccharides (the main components of FTs) and evaluate the appearances of their mixtures.

In this study, we demonstrate that, although the dissolution test showed a delay in drug dissolution owing to XG-FTs, it does not necessarily imply delayed absorption from the gastrointestinal tract. Tomita et al. investigated the effect of XG-FTs on the dissolution of orally disintegrating LVFX tablets as well as their pharmacokinetics in healthy men and reported that the dissolution of these tablets was delayed by XG-FTs with no significant pharmacokinetic differences observed after oral administration in healthy subjects [4]. Therefore, if drug permeability through the gastrointestinal membrane is high, its absorption may not be affected even if the established dissolution standard of the drug product is not satisfied. Therefore, when the dissolution rate of a drug product is delayed by immersion in XG-FTs, it may be necessary to avoid the concomitant use of XG-FTs or confirm that the pharmacokinetics in humans did not change as studied by Tomita et al. Conversely, we plan to observe the appearances of the gel-like precipitate formed by mixing CPFX and FTs and study the effect on absorption. If a gel-like precipitate is formed after mixing FTs with the formulation, its administration should be avoided.

There are two important findings in this study: 1) The delayed dissolution induced by XG-FTs is reduced by film coating but the degree depends on the formulation. It is necessary to investigate the mechanism by which the film coating of a drug product reduces the dissolution delay attributed to XG-FTs; 2) The effect of XG-FTs on drug dissolution depends on the characteristics of the drug substance; the importance of XG-FT interactions with the drug was highlighted. The gel precipitate obtained by mixing XG-FTs and the CPFX formulation caused a clinically relevant decrease in dissolution rate. We confirmed that other agents produced similar gel-like precipitates when immersed in aqueous XG-FTs.

It may be possible that XG-FTs will be used extensively across a wide range of age groups due to the growing number of individuals with dysphagia [2, 15]. Hence, it is important to elucidate the underlying mechanisms of dissolution delay as well as resultant appearance changes owing to the interaction between XG-FTs and pharmaceuticals.

## Conclusion

Hypromellose coating had different effects on LVFX, TFLX, and CPFX film-coated formulations. The dissolution delay due to the aqueous solution of XG-FTs was reduced by the film coating of LVFX and TFLX formulations. On immersion or mixing of CPFX formulations in the XG-FT aqueous solution, a gel-like precipitate was formed, and the dissolution was considerably delayed when compared with other formulations. We have demonstrated that the effect of XG-FT immersion on the dissolution of film-coated fluoroquinolones varies according to the formulation as well as the type of drug substance.

## Data Availability

All data generated or analyzed during this study are included in this published article.

## Abbreviations

FT: Food thickener
XG-FT: Xanthan gum-based food thickener
BCS: Biopharmaceutics classification system
TFLX: Tosufloxacin tosylate monohydrate
LVFX: Levofloxacin hemihydrate
CPFX: Ciprofloxacin hydrochloride monohydrate
JP: Japanese Pharmacopoeia
D15min: 15-min mean dissolution rate

## References

[1] Tomita T, Sakai A, Sato Y, Takanohashi S, Fukui T, Obara M (2019). The use of deglutition aids for Oral Administration of Medication in long-term care health facilities. JJDR..

[2] Lau ETL, Steadman KJ, Cichero JAY, Nissen LM (2018). Dosage form modification and oral drug delivery in older people. Adv Drug Deliv Rev.

[3] Ebata R, Fujita Y, Nakamura A, Harada T (2019). Effect of film coating on xanthan gum solution-induced delays in the disintegration and dissolution of tablets. Jpn J Pharm Health Care Sci.

[4] Tomita T, Yamaguchi A, Nishimura N, Goto H, Sumiya K, Arakawa R (2019). Effect of food thickener and jelly wafer on the pharmacokinetics of levofloxacin orally disintegrating tablets. Heliyon..

[5] Manrique YJ, Sparkes AM, Cichero JA, Stokes JR, Nissen LM, Steadman KJ (2016). Oral medication delivery in impaired swallowing: thickening liquid medications for safe swallowing alters dissolution characteristics. Drug Dev Ind Pharm.

[6] Tomita T, Goto H, Sumiya K, Yoshida T, Tanaka K, Kohda Y (2016). Effects of food thickeners on the inhibitory effect of voglibose oral-disintegrating tablets on post-prandial elevation of blood sugar levels. Yakugaku Zasshi.

[7] Tomita T, Goto H, Sumiya K, Yoshida T, Tanaka K, Kudo K (2017). Effect of food thickener on the inhibitory effect of mitiglinide tablets on post-prandial elevation of blood glucose levels. Dysphagia..

[8] Amidon KS, Langguth P, Lennernas H, Yu L, Amidon GL (2011). Bioequivalence of oral products and the biopharmaceutics classification system: science, regulation, and public policy. Clin Pharmacol Ther.

[9] Wu CY, Benet LZ (2005). Predicting drug disposition via application of BCS: transport/absorption/ elimination interplay and development of a biopharmaceutics drug disposition classification system. Pharm Res.

[10] Onufrak NJ, Forrest A, Gonzalez D (2016). Pharmacokinetic and Pharmacodynamic principles of anti-infective dosing. Clin Ther.

[11] Tsutsui A, Yahara K, Shibayama K (2018). Trends and patterns of national antimicrobial consumption in Japan from 2004 to 2016. J Infect Chemother.

[12] Karlowsky JA, Kelly LJ, Thornsberry C, Jones ME, Evangelista AT, Critchley IA (2002). Susceptibility to fluoroquinolones among commonly isolated gram-negative bacilli in 2000: TRUST and TSN data for the United States. Int J Antimicrob Agents.

[13] Fujitani J, Uyama R, Okoshi H, Kayashita J, Koshiro A, Takahashi K, et al. Japanese Society of Dysphagia Rehabilitation: classificationof dysphagia modified food 2013. JJDR. 2013; https://www.jsdr.or.jp/wp-content/uploads/file/doc/classification2013-manual.pdf. Accessed 22 September 2020.

[14] Doe J. Guideline for bioequivalence studies of generic products. Pharm Med Devices Agency. https://www.nihs.go.jp/drug/be-guide(e)/Generic/GL-E_120229_BE_rev140409.pdf. Accessed 22 July 2020.

[15] Dodrill P, Gosa MM (2015). Pediatric dysphagia: physiology, assessment, and management. Ann Nutr Metab.

